# Influence of joint volume on range of motion after arthroscopic rotator cuff repair

**DOI:** 10.1186/s12891-023-06306-z

**Published:** 2023-03-17

**Authors:** Jung-Han Kim, Young-Kyoung Min, Dae-Yoo Kim, Jun-Ho Park, Young-Chae Seo, Won-Seok Seo

**Affiliations:** 1 Department of Orthopedic Surgery, Inje University Busan Paik Hospital, 70 Bokji-ro, Busanjin-gu, 47392 Busan, Republic of Korea; 2 Geo-In Hospital, Busan, Republic of Korea; 3 Kimhae the Grand Hospital, Gyeongsangnam-Do, Republic of Korea

**Keywords:** Shoulder, Shoulder Joint, Rotator Cuff Injuries, Arthroscopy, Multidetector Computed Tomography, Arthrography

## Abstract

**Background:**

Capsular contracture is a well-known etiology in the primary stiff shoulder; thus capsular contracture and resultant decreased joint volume could lead to postoperative stiffness, which is a commonly reported morbidity after arthroscopic rotator cuff repair (ARCR). The purpose of this study was (1) to quantify the joint volume (total joint volume and each quadrant compartmental volume) using computed tomography arthrography (CTA) and (2) to demonstrate the relationship between joint volume and postoperative range of motion (ROM) after ARCR.

**Materials and methods:**

Eighty-three patients (60 ± 5.11 years, men = 26, women = 57) who had undergone ARCR between January 2015 to December 2020 due to small to medium full-thickness tear and followed by CTA 6 months postoperatively were retrospectively reviewed. An image reconstruction program (3D Slicer, version 4.11.2 software) was used to calculate the joint volume (total joint volume and quadrant compartment joint volumes; anteroinferior, anterosuperior, posterosuperior and posteroinferior). For shoulder ROM, data including scaption (Sc), external rotation on side (ERs), external rotation at 90° (ER90), and internal rotation on back (IRb) were collected 6 months postoperatively. An evaluation of the correlation between joint volume and each shoulder motion was performed.

**Results:**

There were moderate correlations between the total joint volume and each motion (Sc: Pearson coefficient, 0.32, *p* = 0.0047; ERs: Pearson coefficient, 0.24, *p* = 0.0296; ER90: Pearson coefficient, 0.33, *p* = 0.0023; IRb: Pearson coefficient, 0.23, *p* = 0.0336). Among the quadrant compartments, the anteroinferior (Sc: Pearson coefficient, 0.26, *p* = 0.0199; ERs: Pearson coefficient, 0.23, *p* = 0.0336; ER90: Pearson coefficient, 0.25, *p* = 0.0246; IRb: Pearson coefficient, 0.26, *p* = 0.0168) and posterosuperior (Sc: Pearson coefficient, 0.24, *p* = 0.029; ER90: Pearson coefficient, 0.29, *p* = 0.008; IRb: Pearson coefficient, 0.22, *p* = 0.0491) and posteroinferior (Sc: Pearson coefficient, 0.30, *p* = 0.0064; ER90: Pearson coefficient, 0.29, *p* = 0.0072) showed moderate correlations with each shoulder motion.

**Conclusion:**

Total joint volume, anteroinferior compartment joint volume, posterosuperior compartment joint volume and posteroinferior compartment joint volume were related to postoperative ROM after ARCR. Perioperative methods to increase the joint volume, especially the anteroinferior, posterosuperior and posteroinferior parts of the capsule may prevent postoperative stiffness after ARCR.

**Level of Evidence:**

Level III; Retrospective Case-Control Study.

## Introduction

A rotator cuff tear is a common cause of shoulder pain [1, 2]. For the treatment of such tears, arthroscopic rotator cuff repair (ARCR) generally yields successful outcomes due to the development of surgical devices and techniques [3–5]. As the number of ARCRs performed has increased, postoperative complications have also increased [5]. Among these complications, postoperative stiffness, which is a significant factor affecting outcomes, may occur, and its incidence varies from 2.3 to 40% [1, 2, 6].

Postoperative shoulder stiffness is most likely caused by the surgical violation of tissue planes by an arthroscopic instrument or cannula, resulting in contractures of the soft tissue surrounding the articulations, pathologic connections between motion interfaces [5–13]. Among these pathologies, capsular contracture is a well-known etiology (pathologic conditions) that are related to stiff shoulders [5, 8, 10–12]; change in tissue composition stemming from altered connective tissue fibroblast activity leads to capsular contracture and this prevents enough stretching for humeral head movement, therefore causing decreased range of motion (ROM).[11, 14]. Itoi et al., in their evidence base review (Level V study), suggested that shoulder stiffness after rotator cuff surgery is typically global, but posterior capsular contracture is often accentuated [7]. However, because it is difficult to quantify capsular contracture, there is no study evaluating capsular contracture and the relation between capsular contracture and ROM after ARCR. Moreover, There are also not many studies on which portion of capsular contracture is related to specific motion. Volumetric study of glenohumeral joints has been introduced in shoulder instability studies and measurement of joint volume has been used for evaluation of the degree of redundancy in shoulder joints [15–17]. Considering the joint volume has been used to evaluate the degree of capsular redundancy, we think that the joint volume could also be used to evaluate the capsular contracture. Based on the idea, our study design was (1) to quantify the joint volume (total joint volume and quadrant compartment joint volumes), (2) to demonstrate the relationship between joint volume and postoperative ROM, and (3) to demonstrate the relationship between quadrant compartment joint volume (anteroinferior quadrant; AIQ, anterosuperior quadrant; ASQ, posterosuperior quadrant; PSQ and posteroinferior quadrant; PIQ) and postoperative ROM. The study hypothesis was that the joint volume (total joint volume and quadrant compartment joint volumes) is related to postoperative ROM after ARCR.

## Materials and methods

### Patient selection

**Fig. 1:**
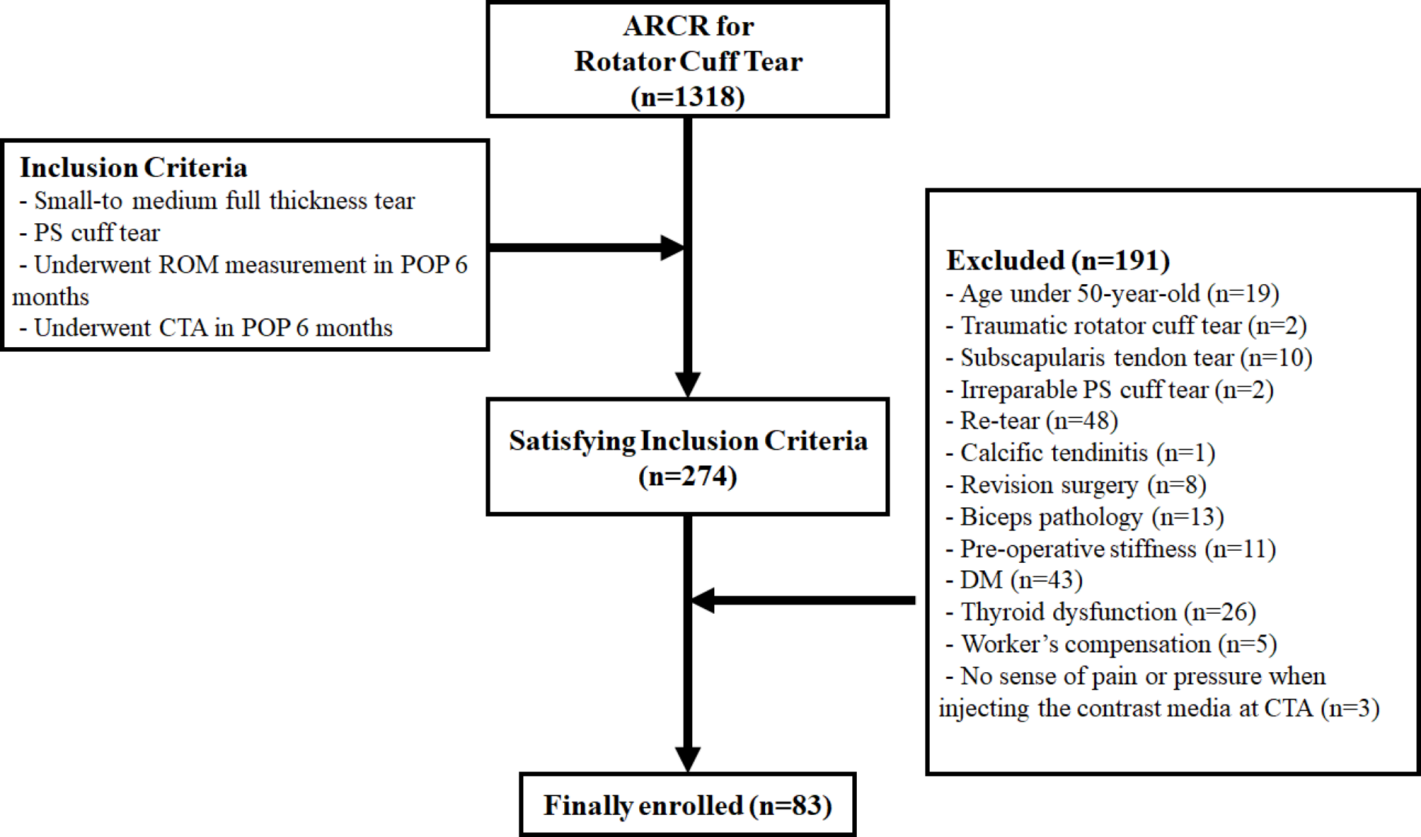
Patient flow chart showing the inclusion and exclusion criteria used in this study

After obtaining approval from the institutional review board, we retrospectively reviewed 1,318 patients who had undergone ARCR for a rotator cuff tear between January 2015 and December 2020. Among them, patients with small to medium (less than 25 × 25 mm anterior-posterior and medial-lateral diameter) full-thickness tears of the posterior-superior (PS) cuff were included. Moreover, patients who had undergone ROM measurement and postoperative shoulder computed tomography arthrography (CTA) 6 months postoperatively were included. The exclusion criteria were as follows: age younger than 50 years (to analyze within similar age groups); traumatic rotator cuff tear, subscapularis tendon tear, irreparable PS cuff tear, and re-tear, calcific tendinitis, revision surgery, biceps pathology, preoperative stiffness, diabetes mellitus (DM) [1, 5, 7, 12] thyroid dysfunction [5, 7] (to exclude medical conditions affecting postoperative stiffness); receipt of worker’s compensation [1, 5, 7] (which may also affect postoperative stiffness); and those who received all 20 mL of contrast medium [18] into the shoulder joint during CTA (to simulate identical filling pressure). Among the 274 patients who satisfied the inclusion criteria, 83 patients were finally selected after applying the exclusion criteria (Fig. 1).

### Surgical method

All surgeries were performed by the one surgeon using a standardized technique. After receiving general anesthesia, patients were positioned in a beach chair at a 70° angle. The arthroscopic evaluation of all associated intra-articular lesions was performed via a standard posterior portal. The anterior portal was created just above the superior margin of the subscapularis tendon and used as a working portal. For PS cuff tears, bone preparation was performed in the footprint area with a shaver and electrocautery device (Edge®, Bipolar Arthroscopic RF System; ConMed Linvatec, Largo, FL) followed by a single-row repair using a 4.5-mm bio- Corkscrew® suture anchor (Arthrex, Naples, Florida, USA). Capsular release was not performed in all patients.

### CTA imaging protocols

All patients were informed about the procedure, timing, and possible complications of CTA. After local anesthesia on the lower one-third level of the glenohumeral joint, the joint was punctured with a spinal needle to deliver a small amount of iodine contrast medium (Ultravist), verifying that the needle tip was positioned inside the joint space. Under fluoroscopic guidance, contrast medium was used to confirm intra-articular location of needle tip. With gentle and progressive injection, flow of contrast medium away from the needle tip and opacification of the joint space confirm adequate position. Then, a solution containing 12.5 mL of saline, 6 mL of iopromide (Ultravist; Bayer Healthcare Pharmaceuticals), and 1.5 mL of mepivacaine (Mevan, 20 mg/mL per vial; Hanlim) was slowly injected by one radiologist until the patient felt pain and pressure. The patients were placed in the supine position in a CT scanner with the affected arm adducted and the head turned to the unaffected side. The scan proceeded from the superior aspect of the acromioclavicular joint to several centimeters inferior to the inferior angle of the scapula. CTA was performed using three different machines: Somatom Drive (180 mA; 120 kV; 1-mm thickness), Somatom Definition AS (117 mA; 140 kV; 1-mm thickness), and Somatom Perspective (110 mA; 130 kV) (all by Siemens) with a slice thickness of 1 mm.

### Image reconstruction

**Fig. 2:**
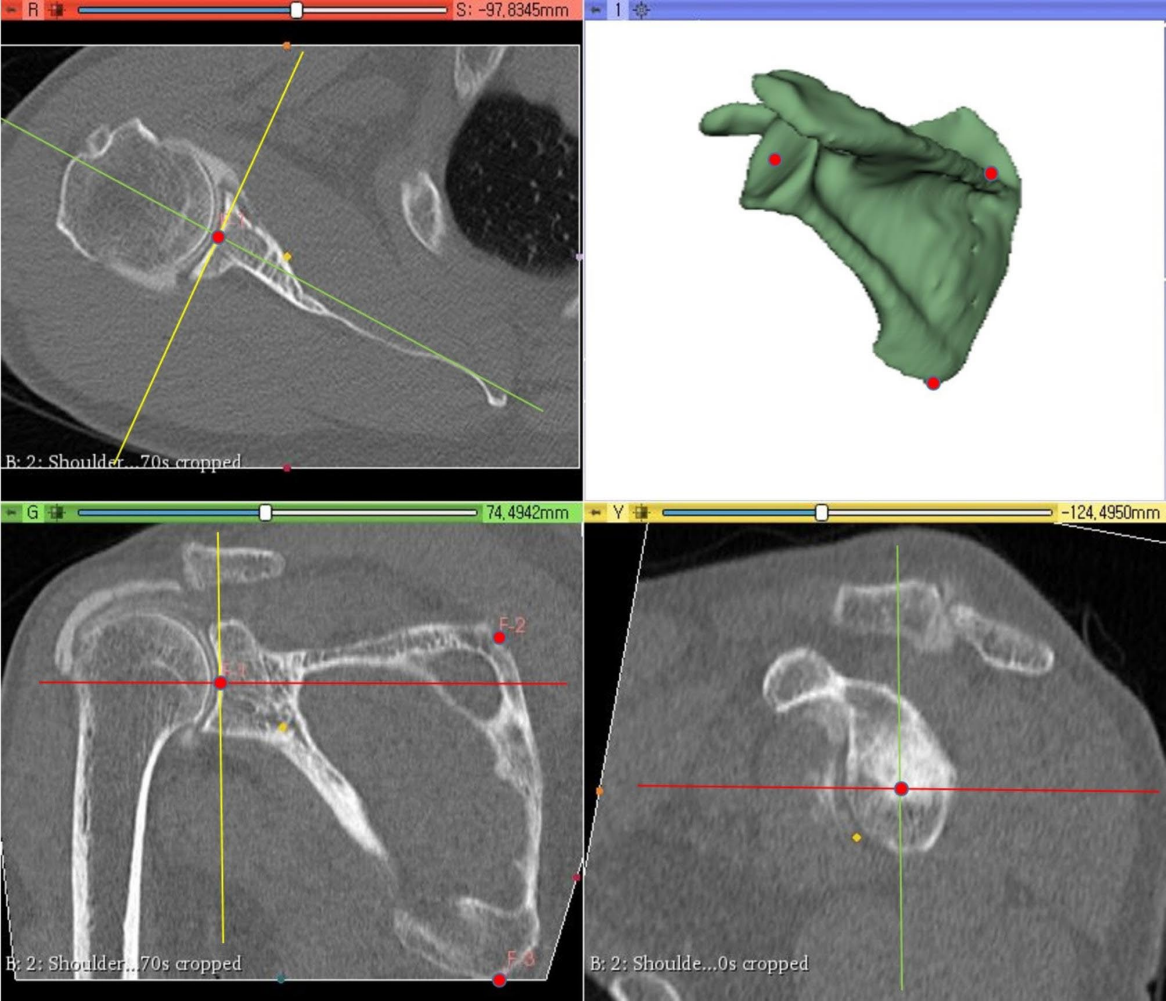
Registration of three scapular landmarks (angular inferior, trigonum scapulae, and glenoid center) as fiducials before reconstruction. Scapular plane (green plane in the lower left image, green line in the upper left and lower right images): plane that passes through the three scapular landmarks. Axial plane (red plane in the upper left image, red line in the lower right and lower left images): plane that is perpendicular to the scapular plane. Sagittal plane (yellow plane in the lower right image, yellow line in the upper left and lower left images): plane that is orthogonal to the scapular and axial planes

CTA images of the patients were saved as DICOM files, and reconstruction to 3D images was performed using an image reconstruction program (3D Slicer, version 4.11.2 software) [15, 19]. The current study aimed to use not only the total joint volume but also four different quadrant compartments of the joint, which is divided by the scapular plane; therefore, modification of the coordinate plane was warranted prior to joint volume measurement. Three scapular landmarks were first registered as fiducials: the angular inferior, trigonum scapulae, and glenoid center of the scapula (Fig. 2). Then, a plane passing through the three landmarks was set as the scapular plane [13, 15]. With the scapular plane as a reference, the coordinate space of the image was modified, with the scapular plane itself as a coronal plane, the perpendicular plane as an axial plane, and a plane orthogonal to both the scapular plane and axial plane as a sagittal plane.

**Fig. 3:**
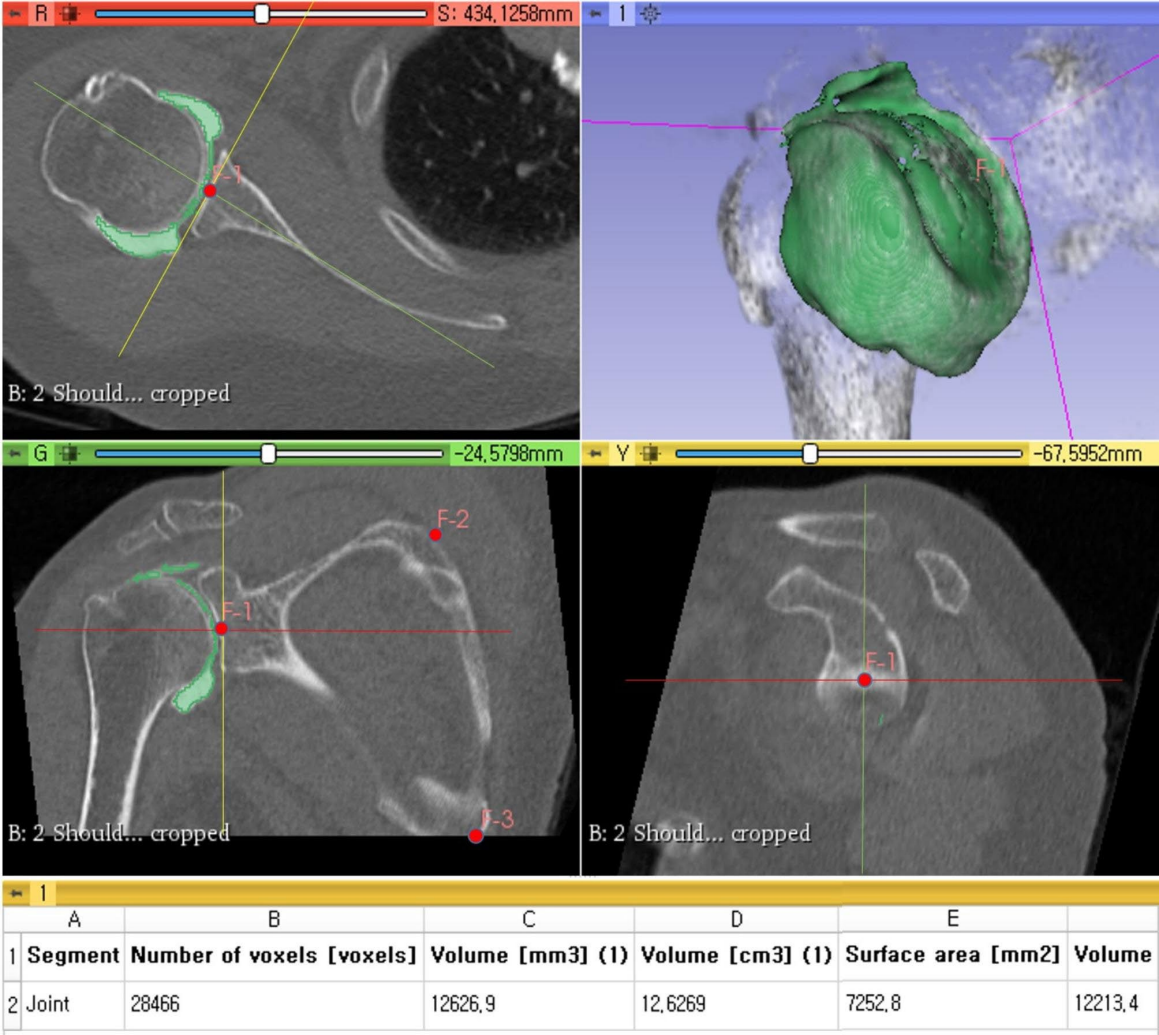
Separation of joint portion of image from others in semi-automated manner. If additional modification was needed, further manual erasing and segmentation were performed (upper right, lower left, and lower right images). When separation was performed appropriately, a 3D image of the segmented joint was generated (upper right image). The total joint volume was then calculated via the “Segment statistics” module

Thereafter, the contrast media of the joint was separated from bony structures using the “Grow from seeds” effect, which semi-automatically reconstructs the targeted anatomical structure based on the registered “seeds”. The semi-automated segmentation was followed by a manual erase feature to remove any excessively segmented portions or by manual segmentation for parts that required additional segmentation (Fig. 3). After separation was appropriately performed, a 3D image of the segmented joint was generated (Fig. 3). The total joint volume was calculated by applying the “Segment statistics” module using this image (Fig. 3).

In the scapular sagittal plane, the segmented joint image could be further divided into anterior and posterior halves using the scapular coronal plane and into superior and inferior halves using the scapular axial plane. The center of the glenoid was set as the common origin of the coordinate plane. With center of the glenoid as the center of the clock, we defined 6 to 9 h as AIQ, 9 to 12 h as ASQ, 12 h to 3 h as PSQ, 3 to 6 h as PIQ. (In the remainder of this article, we note clockface positions as ‘‘h’’; e.g., 3 h refers to the 3-o’clock position.)

**Fig. 4:**
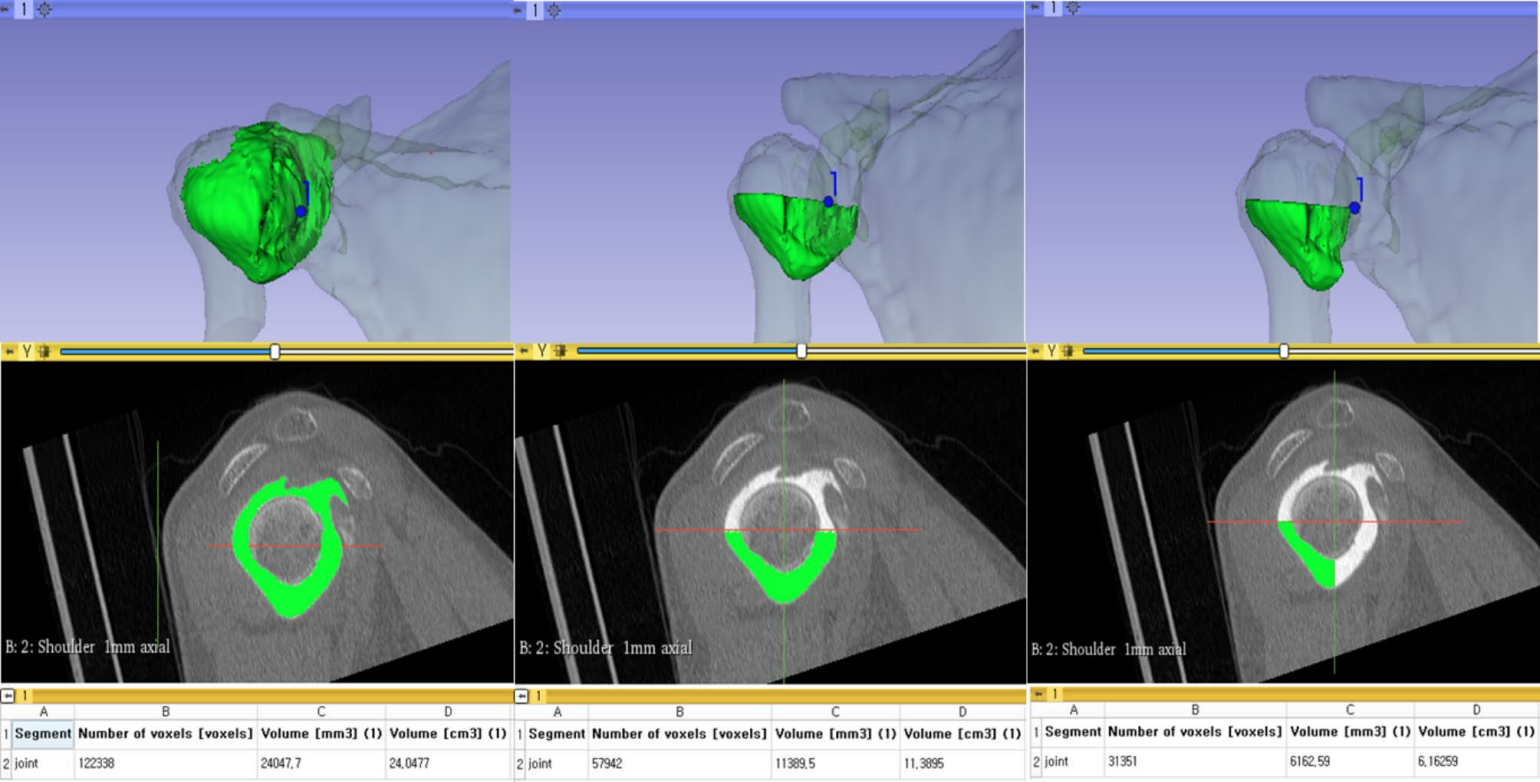
Further separation of the total joint volume into each quadrant compartment joint volume performed in the scapular sagittal plane. The figure shows that the joint portion was separated into inferior compartment and then posteroinferior joint portions. Thereafter, calculation of the separated joint volume was performed via the “Segment statistics” module

The specific quadrant volume was reconstructed by discarding the other parts using the “Scissors effect” located in the “Segment editor” module (Fig. 4). Then, the same procedure using the “Segment statistics” module was performed to calculate the volume of each compartment.

To assess the interobserver reliability, two separate orthopedic surgeons measured the total joint volume and each quadrant compartment volume for all patients. For intraobserver reliability, a single surgeon (with 5 years of experience) performed the measurements twice with the same images at a 2-month interval. To evaluate the consistency among the raters, intraclass correlation coefficients (ICCs) were calculated. Values less than 0.5 indicate poor reliability, those between 0.5 and 0.75 indicate fair reliability, those between 0.75 and 0.9 indicate good reliability, and those greater than 0.9 indicate excellent reliability.

### ROM assessment

Measurement of patients’ passive ROM 6 months postoperatively was performed by one independent medical examiner using a goniometer. Four different motions were evaluated: scaption (Sc), external rotation on side (ERs), external rotation at 90° (ER90), and internal rotation on back (IRb).

The ROM patient data were reviewed and evaluated 6 months after ARCR via electric medical records. To facilitate the statistical analysis, IRb values were converted into contiguously numbered groups [20]: buttock for 1, L5 to L1 for 2 to 6, and T12 to T1 for 7 to 18.

### Rehabilitation method

All patients underwent identical postoperative physical therapy regimens. A customized abduction pillow brace was placed on the patient’s shoulder immediately after the surgery. Passive immobilization was performed for 6 weeks. Thereafter, the brace was removed, and active mobilization with coordination training was performed for a further 4 weeks. Finally, specific progressive resistance exercises were prescribed. Patients begin progressive resistance exercise with the Thera-Band® (HCMHygenic Corp, Batu Gajah, Malaysia). With the arm tucked close to the body, use rubber tubing to provide resistance to internal rotation of the arm. Turn around to use the tubing to provide resistance to external rotation of the arm.

### Statistical analysis

A specialized statistician from the author’s institution performed the statistical evaluation using IBM SPSS Version 25.0 (IBM Corp., Armonk, NY, USA). Pearson’s correlation analysis was used to evaluate the relationship between joint volume (total joint volume and quadrant compartment joint volumes) and each shoulder motion. A p-value less than 0.05 was considered statistically significant.

## Results

The epidemiological information, total joint volume, quadrant compartment joint volume, and ROM data of the study participants are summarized in Table 1. The reliability of the measurements for the total joint volume and each quadrant compartment joint volume was excellent, ranging from 0.767 to 0.917 for interobserver reliability and from 0.777 to 0.937 for intraobserver reliability.

**Table 1:** Patients’ demographic data

Demographic Data	Mean ± Standard Deviation (Range)
Age, year	59.59 ± 5.11 (50–69)
Height, cm	159.11 ± 7.99 (133–177)
BMI, kg/m^2^	23.95 ± 3.09 (15–32.1)
Sex, male : female	26 : 57
Tear size (AP), mm	11.80 ± 4.95 (2.5–25)
Tear size (ML), mm	11.56 ± 5.48 (3–26)
Scaption, degree	159.71 ± 25.23 (80–180)
External rotation on side, degree	47.21 ± 17.35 (10–90)
External rotation on 90°, degree	76.58 ± 16.14 (20–90)
Internal rotation on back, point	7.07 ± 3.67 (1–18)
Total joint volume, mL	10.38 ± 3.14 (3.14–10.38)
Vol.AIQ, mL	2.17 ± 1.1 (0.53– 5.94)
Vol.ASQ, mL	2.28 ± 0.85 (0.84–4.40)
Vol.PSQ, mL	2.60 ± 1.23 (0.86–7.12)
Vol.PIQ, mL	3.14 ± 1.34 (0.60– 8.47)

^a^BMI, body mass index; AP, anterior-posterior; ML, medial-lateral; Vol.AIQ, anteroinferior quadrant joint volume; Vol.ASQ, anterosuperior quadrant joint volume; Vol.PSQ, Posterosuperior quadrant joint volume; Vol.PIQ, posteroinferior quadrant joint volume

### Correlations between total joint volume and shoulder motions

**Fig. 5:**
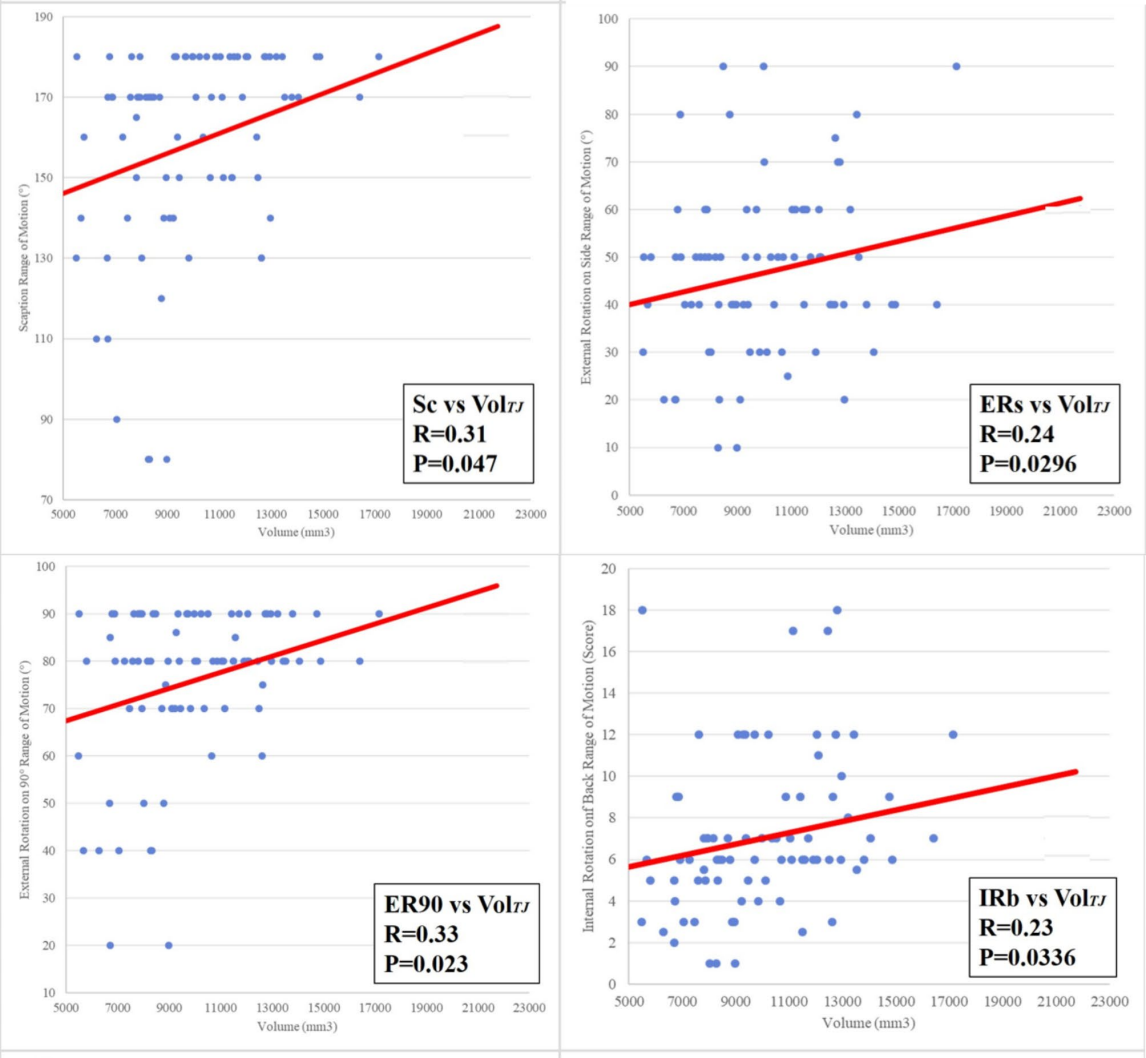
Scatter matrix of the relationship between the total joint volume (Vol.TJ) and each shoulder motion (Sc: scaption, ERs: external rotation on side, ER90: external rotation at 90°, IRb: internal rotation on back). The Vol.TJ showed a moderately positive correlation with each of the four shoulder motions

The correlation analysis between total joint volume and each shoulder motion is shown in Fig. 5. Total joint volume showed a moderately positive correlation with each shoulder motion (Sc: Pearson coefficient, 0.32, *p* = 0.0047; ERs: Pearson coefficient, 0.24, *p* = 0.0296; ER90: Pearson coefficient, 0.33, *p* = 0.0023; IRb: Pearson coefficient, 0.23, *p* = 0.0336).

### Correlations between quadrant compartment joint volumes and shoulder motions

The correlation analysis between quadrant compartment joint volumes and each shoulder motion is shown in Table 2. The AIQ compartment joint volume showed a moderately positive correlation with each shoulder motion (Sc: Pearson coefficient, 0.26, p = 0.0199; ERs: Pearson coefficient, 0.23, p = 0.0336; ER90: Pearson coefficient, 0.25, p = 0.0246; IRb: Pearson coefficient, 0.26, p = 0.0168). Moreover, the PSQ compartment joint volume showed a moderately positive correlation with specific shoulder motion (Sc: Pearson coefficient, 0.24, p = 0.029; ER90: Pearson coefficient, 0.29, p = 0.008; IRb: Pearson coefficient, 0.22, p = 0.0491). The PIQ compartment joint volume showed a positive correlation with specific shoulder motion (Sc: Pearson coefficient, 0.30, p = 0.0064; ER90: Pearson coefficient, 0.29, p = 0.0072).

**Table 2:** Correlation Between Each quadrant Compartment Joint Volume and Each Shoulder Motion

Motion		Vol.AIQ	Vol.ASQ	Vol.PSQ	Vol.PIQ
Sc					
	Pearson Coefficient	0.26	0 0.00	0.24	0.30
	p-value	0.0199*	0.9974	0.029*	0.0064*
ERs					
	Pearson Coefficient	0.23	0.09	0.18	0.16
	p-value	0.0336*	0.4319	0.1061	0.1611
ER90					
	Pearson Coefficient	0.25	0.03	0.29	0.29
	p-value	0.0246*	0.7961	0.008*	0.0072*
IRb					
	Pearson Coefficient	0.26	-0.07	0.22	0.18
	p-value	0.0168*	0.548	0.0491*	0.0976

^a^Sc, Scaption; ERs, External Rotation on Side; ER90, External Rotation on 90°; IRb, Internal Rotation on Back; Vol.AIQ, anteroinferior quadrant joint volume; Vol.ASQ, anterosuperior quadrant joint volume; Vol.PSQ, posterosuperior quadrant joint volume; Vol.PIQ, posteroinferior quadrant joint volume

*P value < 0.05

**Fig. 6:**
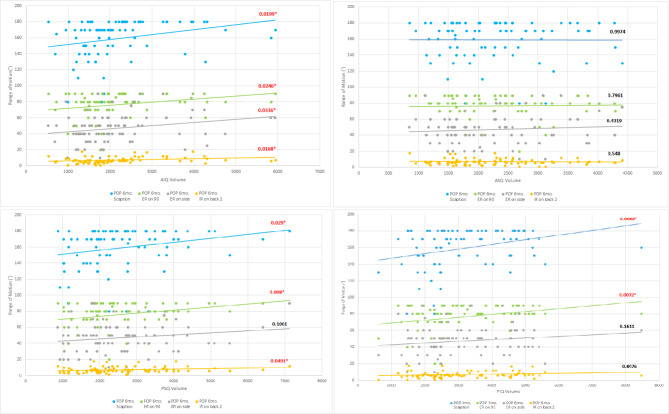
Scatter matrix of the relationship between each quadrant compartment joint volumes (Vol. anteroinferior, Vol.anterosuperior, Vol.Posterosuperior, Vol.Posteroinferior) and each shoulder motion (Sc: scaption, ERs: external rotation on side, ER90: external rotation at 90°, IRb: internal rotation on back). The Vol. anteroinferior, Vol.Posterosuperior, Vol.Posteroinferior showed a moderately positive correlation with each of the four shoulder motions

**Fig. 7:**
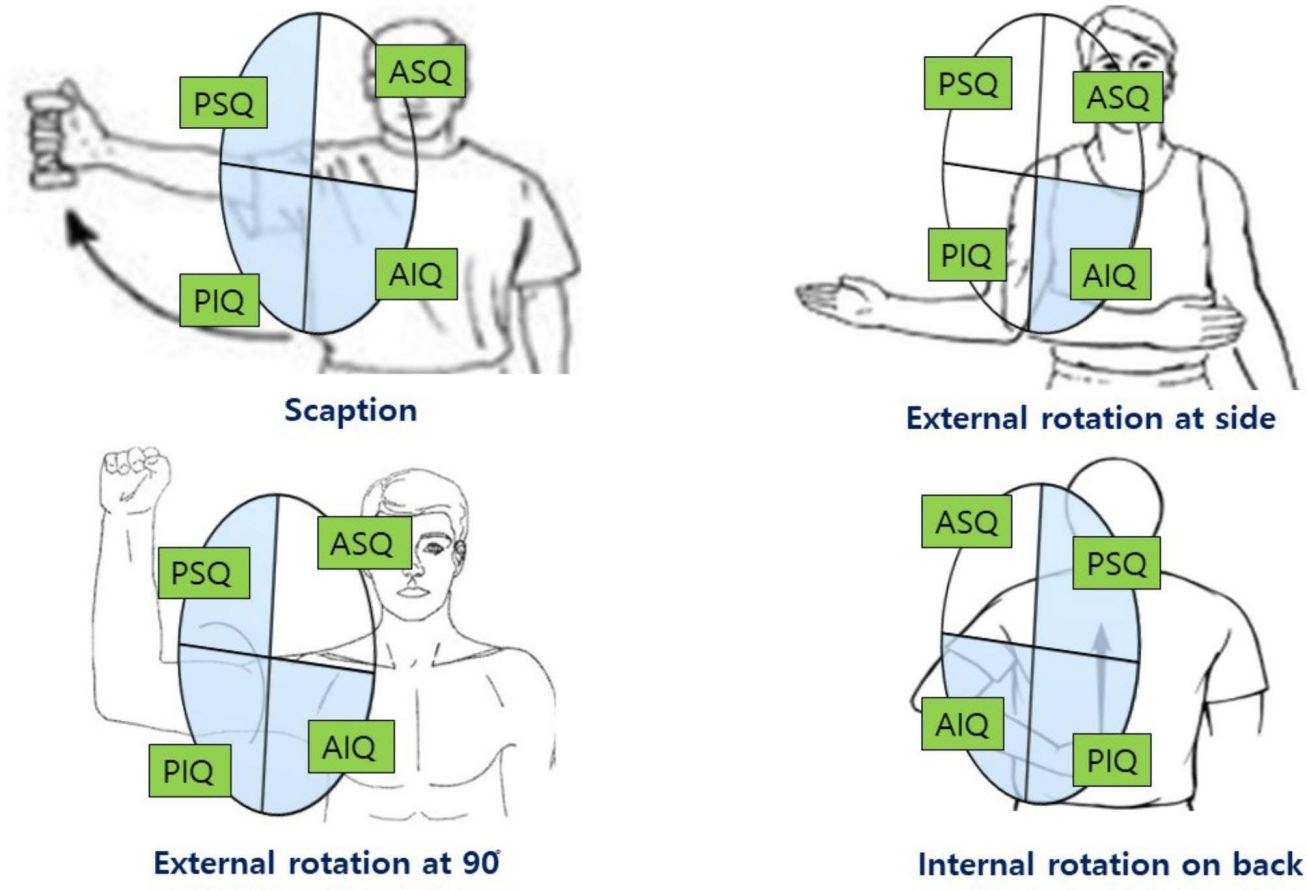
A schematic diagram showing the shoulder joint volume affected by postoperative tightening in a specific movement. Among the quadrant (AIQ: Anteroinferior quadrant, ASQ: Anterosuperior quadrant, PSQ: Posterosuperior quadrant, PIQ: Posterosuperior quadrant), the affected area in specific joint motion are filled in blue

This results expressed as scatterplots in Fig. 6. In addition, tightness in specific directions according to specific capsular quadrant was schematically illustrated in Fig. 7.

## Discussion

The main findings of this study were that (1) there was positive correlation between the total joint volume and each shoulder motion and (2) there were positive correlations between AIQ, PSQ and PIQ compartment joint volume and spectific shoulder motion.

Postoperative stiffness after ARCR is commonly reported morbidity after ARCR and concerns to shoulder surgeons due to inferior functional outcome when developed.[1, 2, 6]. Although the pathophysiology of stiffness after ARCR is not well understood, postoperative ROM is affected by several factors, such as capsular contracture, contracture or atrophy of the rotator cuff itself, and adhesions within the extra-articular glenohumeral motion interface.[7, 12]. However, there is a lack of logical development in the correlation whether the decrease in range of motion was caused by surgery. For proving a cause-effect relationship between range of motion and joint volume after rotator cuff repair, we should have evaluated the relation between change of range of motion and change of joint volume. But, the test to obtain the volume of the shoulder joint (In our study; shoulder CT arthrography) are not performed usually and routinely in the pre-op patients. So we designed the study for evaluating postoperative shoulder joint volume, which could reflect capsule contraction after rotator cuff repair. Our study goal was not to find out the cause of the decrease in joint volume after surgery by comparing the range of joint motion before and after surgery. Several investigators tried to quantitatively measure pathologic regions in MRI images for evaluating capsular contracture; the width, depth, and height of the axillary recess, dimension of rotator interval and the glenohumeral distance [21–23]. However, these studies evaluated pathologic regions in 2D images, which may not accurately reflect the status of the capsular contracture. Other authors measured the capsular volume of the glenohumeral joint according to the volume of fluid injected into the capsule with or without pressure measurement.[1, 12, 24]. Even though these methods are excellent for evaluating capsular contracture, procedures such as fluid injection, volume and/or pressure measurement are not easily applied to patients during follow-up after operation. In the current study, we calculated the volume of the shoulder joint using CTA. CTA has been routinely performed to patients who underwent ARCR at postoperative 6 months in our hospital for evaluating cuff continuity, and selection bias can be reduced. Using 3D Slicer software which has been used in a variety of medical studies, [15, 17, 19] and its accuracy and efficiency in 3-dimensional segmentation and analysis have been well described, we could measure capsular volume through CTA DICOM files.

Decreased total joint volume in a primary stiff shoulder is well documented in previous studies [23, 25, 26]. However, previous studies used comparative analyses and reported decreased joint volume in the stiff group. Therefore, the relation between joint volume and ROM could not be evaluated. Moreover, the subjects in the previous study were primary stiff shoulder patients. Primary shoulder stiffness and postoperative stiffness after ARCR are considered to be different disease entities due to their different natural courses, [6, 9, 19, 27, 28] therefore, the results of the previous studies could not be applied to postoperative stiffness after ARCR. In the current study, we performed a correlation analysis between total joint volume and postoperative ROM after ARCR. The correlation analysis between total joint volume and each shoulder motion showed a moderately positive correlation with each shoulder motion (Sc: Pearson coefficient, 0.32, *p* = 0.0047; ERs: Pearson coefficient, 0.24, *p* = 0.0296; ER90: Pearson coefficient, 0.33, *p* = 0.0023; IRb: Pearson coefficient, 0.23, *p* = 0.0336). This result implies that total joint volume is related to the postoperative ROM and that a decrease in total joint volume could lead to postoperative stiffness. Therefore, procedures to increase the total joint volume such as selective capsulectomy during operation, posterior capsular stretching exercise after operation etc., are crucial to increase the postoperative ROM, and this can further lead to the prevention of postoperative stiffness. Although the usefulness of capsulectomy and early rehabilitation remain controversial, [1, 6, 9, 14] based on our results, procedures that increase total joint volume may help prevent postoperative stiffness from a clinical perspective.

In the current study, we could easily separate specific areas of joint volume (anteroinferior, anterosuperior, posterosuperior and posteroinferior quadrant) using 3D Slicer software and evaluate the relation between specific area joint volume and ROM after ARCR. Our study showed that anterior compartment volume (especially AIQ) was related to ERs, posterior compartment joint volume (PSQ & PIQ) related to IRb, and inferior compartment joint volume (AIQ & PIQ) related to Sc. These findings were similar to the results of previous studies [11, 29–31]. In our study, Interestingly, AIQ joint volume were related to all shoulder motion and inferior compartment volume (AIQ & PIQ) were also related to ER at 90 and IRb. In addition, posterior compartment volume (PSQ & PIQ) was related to not only IRb but also Sc and ER at 90. Even though we cannot delineate the cause of these findings from current study, this result may be due to methodological differences. Previous studies reproduced joint contracture by selective capsular plications or thermal impacts for ligament shortening to change the specific joint volume and then measured passive range of motion. Whereas, we used shoulder CT arthrography to obtain specific joint volume postoperatively and measured the ROM of patients. In our study, positive correlations between posterior half and inferior half compartment joint volume (AIQ & PSQ & PIQ) and specific shoulder motion are clinically meaningful. Given that the posterior and inferior capsular contracture is often accentuated after ARCR and posterior half and inferior half compartment joint volume are related to specific shoulder ROM, [7] efforts to increase the posterior half and inferior half compartment joint volume such as selective capsulectomy during operation and posterior capsular stretching exercise after operation may be useful in the prevention and treatment of postoperative stiffness.

There are several limitations in the current study. (1) Among 1318 consecutive patients during the study period, 274 patients were included after applying the inclusion criteria. During applying inclusion criteria, only 20% patients were selected. In addition, among 274 patients, 83 patients were selected as final subjects after applying the exclusion criteria. Thus, this study may have a selection bias. However, we wanted to evaluate the relation between joint volume and ROM after arthroscopic repair of small to medium sized rotator cuff tears. In addition, we also excluded patients who had factors that are known to affect postoperative stiffness. Considering that the final subjects are selected after applying inclusion and exclusion criteria among consecutive patients, selection bias may be minimized. (2) Evaluation of the patients’ ROM was done at 6 months postoperatively, which can be a short observation period for defining postoperative stiffness. Although, goal of our study was not to evaluate joint stiffness after surgery, but to assess the relationship between joint volume and specific range of motion after surgery. Therefore, it is not necessary to evaluate after the stiffness is completely resolved after surgery. In addition, as a retrospective study, we have been conducting CT arthrography for six months after surgery routinely because “naturally resolving” postoperative stiffness patients mostly shows improvement of ROM within 6 months and further observation period makes no difference because tendon stops healing and starts to remodel at this time [1, 20, 28, 29]. (3) Although we obtained excellent interobserver reliability (Ranging from 0.767 to 0.917) and intraobserver reliability (0.777 to 0.937), measurement error could not completely be excluded because the joint volume was measured in semi-automated manner. (4) For the evaluation of the degree of capsular contracture, measuring the intra-articular pressure is more accurate method than measuring joint volume. However, it is very difficult to measure intra-articular pressure for every follow-up patients and to measure pressure of specific joint compartment. Therefore, we used joint volume to evaluate the degree of capsular contracture. However, joint volume depends on the expansion behavior of the capsule which is not linear but logarithmic. To overcome this problem, we stopped injection of contrast media at the time patient felt pain and pressure during CTA. Furthermore, we excluded the patient who did not felt pain or pressure and received all 20 mL of contrast media in this study. (5) Clinical scores are not assessed in this study. Study about the influence of joint volume on clinical scores may have induced another clinically meaningful results and further study regarding this issue seems to be useful.

In conclusion, the total joint volume showed positive correlation with postoperative ROM after ARCR. Specifically, among quadrant compartment joint volumes, posterior half and inferior half compartment joint volume (AIQ, PSQ and PIQ joint volumes) were related to ROM after ARCR. Considering its frequent incidence and effect on poor clinical outcome, preventing the postoperative stiffness after ARCR is an important issue and perioperative methods to increase the total joint volume, especially the posterior or inferior part of the capsule seems to be useful.

## Data Availability

The datasets used and/or analysed during the current study available from the corresponding author on reasonable request.
